# Prescription and Underprescription of Clozapine in Dutch Ambulatory Care

**DOI:** 10.3389/fpsyt.2018.00231

**Published:** 2018-06-11

**Authors:** Yvonne C. van der Zalm, Fabian Termorshuizen, Peter F. Schulte, Jan P. Bogers, Machteld Marcelis, Iris E. Sommer, Jean Paul Selten

**Affiliations:** ^1^Department of Psychosis Research, Rivierduinen Psychiatric Institute, Leiden, Netherlands; ^2^Department of Psychiatry and Neuropsychology, Maastricht University Medical Center, School for Mental Health and Neuroscience, Maastricht, Netherlands; ^3^Dutch Clozapine Collaboration Group, Castricum, Netherlands; ^4^Mental Health Service Noord-Holland Noord, Alkmaar, Netherlands; ^5^Research Department, Institute for Mental Health Care Eindhoven, Eindhoven, Netherlands; ^6^Department of Neuroscience and Department of Psychiatry, University Medical Center Groningen, Groningen, Netherlands

**Keywords:** clozapine, psychotic disorders, outpatient care, prescription rates, treatment resistance

## Abstract

**Purpose:** To our knowledge, no study has examined in a structured way the extent of underprescription of clozapine in ambulatory patients with Non-Affective Psychotic Disorder (NAPD). In the Netherlands, psychiatric care for such patients is provided by Flexible Assertive Community Treatment (FACT) teams and by early intervention teams. In 20 FACT teams and 3 early intervention teams we assessed the proportion of patients who: use clozapine (type 1 patients), previously used this drug (type 2), have an unfulfilled indication for this drug, by type of indication (type 3), or were at least markedly psychotic, but had not yet received two adequate treatments with other antipsychotic drugs (type 4). We expected to find major differences between teams. To rule out that these differences are caused by differences in severity of psychopathology, we also calculated the proportions of patients who use clozapine given an indication at any time (number of type 1 patients divided by the sum of type 1, 2, and 3 patients).

**Materials and methods:** The nurse practitioner of each team identified the patients already on clozapine. Next, using a highly-structured decision tree, the nurse practitioner and psychiatrist assessed whether the remaining patients had an indication for this drug. Indications were treatment-resistant positive symptoms, tardive dyskinesia, aggression and suicidality. The severity of positive symptoms was determined using the Clinical Global Impression-Schizophrenia Scale (CGI-SCH).

**Results:** In the participating FACT-teams 2,286 NAPD patients were assessed. The range among teams in proportions was: type 1: 8.8–34.7% (mean: 23.0%), type 2: 0–8.2% (mean: 3.5%), type 3: 1.7–15.6% (mean: 6.9%), type 4: 1.8–16.3% (mean: 8.6%). The range in proportions of patients using this drug given an indication was 49.0–90.9% (mean: 68.8%). These figures were lower in early intervention teams.

**Conclusions:** The proportion of patients in FACT-teams who have an unfulfilled indication for clozapine is 6.9%. There were considerable differences between teams with respect to this proportion. Almost a third of the outpatients had at any time an indication for clozapine. If one takes type 4 patients into account, this proportion may be higher.

**Registration number:** NTR5135 http://www.trialregister.nl/trialreg/index.asp

## Introduction

Despite the general idea that clozapine is underutilized, little research has been done into the extent of this problem. The main indication for this drug according to guidelines is treatment-resistant schizophrenia, the prevalence of which has been estimated at about 20–30% (1), but exact numbers are unknown. This uncertainty is not only caused by a scarcity of pertinent studies, but also by the absence (until recently) of consensus on criteria to define treatment-resistant schizophrenia (2). To illustrate this, Juarez-Reyes et al. (3) found that the proportion in a population of outpatients was 12.9% with a stringent definition and 42.9% with a broad definition. A Danish register-based study among outpatients with a diagnosis of schizophrenia found a prevalence of 24.7 or 48.2% (4), depending on the definition of a proxy for treatment-resistant schizophrenia: (1) at least two different periods of antipsychotic use and one hospitalization within 18 months and (2) patients treated with polypharmacy for at least 90 days. No information on adherence and symptom severity was available, which precludes an exact assessment of treatment-resistant schizophrenia.

Furthermore, there are also other indications for clozapine, as it has been found to decrease tardive dyskinesia (5), acute extrapyramidal symptoms (i.e., parkinsonism, acute dystonia, and akathisia) (6), aggression (7–9), suicidality (10, 11), and substance abuse (12). The prevalence of these other indications is even more uncertain.

Studies of clozapine prescription rates show large international differences (13–15). In the Netherlands, the proportion of patients with Non-Affective Psychotic Disorder (NAPD) using this drug is unknown. The vast majority of them is treated on an ambulatory basis by Flexible Assertive Community Treatment (FACT) teams and in some regions also by early intervention teams. FACT-teams take care of patients with a severe mental illness and are called “flexible” because they intensify treatment when the patient is in a crisis, with the aim to prevent hospitalization (16). These teams are responsible for a certain area and treat approximately 200 outpatients, most of whom with NAPD. Some institutes deploy specialized early intervention teams to treat patients in the first years after onset of psychosis. These teams work in the same way as the FACT-teams, but they spend more time on diagnosing patients and providing psychoeducation. Their caseloads may be smaller and the patients are younger. After a maximum of 5 year, treatment will be continued by a FACT-team. In general, in every FACT or early intervention team there is a psychiatrist, but only part of the teams have a nurse practitioner associated with it.

Summarizing, little is known about the magnitude of underprescription of clozapine in outpatients with NAPD in the Netherlands. We set out to examine in a structured way rates of prescription and underprescription in FACT teams and in early intervention teams. We developed a decision tree with criteria for an indication for clozapine. Our definition of treatment resistance differs only slightly from the consensus guideline that was published shortly after we collected our data (17). Since in our experience there are major regional and personal differences in adherence to guidelines regarding clozapine prescription, we expected considerable differences between teams in proportions of patients using this drug and patients with an unfulfilled indication. In order to exclude the possibility that these differences are solely caused by differences in the severity of psychopathology, we calculated for each team the proportion of patients who use clozapine given an indication at any time.

The aims of this study were to determine prescription rates and the extent of underprescription of clozapine in outpatients with NAPD. We therefore assessed the proportions of patients who (i) currently use clozapine (type 1 patients); (ii) had used this drug and subsequently discontinued it (type 2 patients); (iii) have an indication for this drug but have never used it, by type of indication (type 3 patients); (iv) were at least markedly psychotic, but had not yet received two adequate treatments with antipsychotic drugs (type 4 patients); (v) currently use clozapine among patients with an indication for this drug at any time (number of type 1 patients divided by the sum of type 1, type 2, and type 3 patients).

## Materials and methods

### Study design and setting

This study reports the results of baseline measurements preceding a randomized controlled trial to assess the safety of the deployment of nurse practitioners to start patients on clozapine. Therefore, in this study, only teams with a nurse practitioner associated with it were included. Twenty FACT teams and three early intervention teams, from four Dutch psychiatric institutes, participated. Each institute deploys several teams, housed in the same building or at miles distance from each other. In all participating teams, the psychiatrist was responsible for the prescription of antipsychotic drugs. Data was collected from July 2015 to May 2016.

### Measures

According to Dutch guidelines (18, 19), clozapine is indicated for patients with a diagnosis of schizophrenia or schizoaffective disorder, who suffer from (1) treatment-resistant positive or negative symptoms, (2) severe aggressive behavior, (3) persistent suicidal behavior, (4) tardive dyskinesia, (5) treatment-resistant acute extrapyramidal symptoms, and (6) alcohol or drug abuse (18). However, since the current evidence to support the use of clozapine for treatment-resistant negative symptoms or substance abuse is insufficient, this study did not regard these features as indications for clozapine.

In order to structure the assessment of an indication for clozapine, we developed a decision tree (see Appendix). Positive symptoms were scored using the Clinical Global Impression-Schizophrenia Scale (CGI-SCH), a simple instrument, appropriate for use in observational studies (20). The researchers who developed this instrument, reported that the correlation coefficient between the CGI-SCH for positive symptoms and the PANSS score was 0.86 and the interrater reliability was high (intraclass correlation coefficient, ICC = 0.82). Possible scores for positive symptoms are “normal, not ill” (1), “minimally ill” (2), “mildly ill” (3), “moderately ill” (4), “markedly ill” (5), “severely ill” (6), and “among the most severely ill” (7) (20) (see Appendix for a more detailed description of the scores). We defined treatment-resistance of positive symptoms as the persistence of at least markedly severe positive symptoms (score 5 or higher), despite adequate treatment. Adequate treatment was defined as having used two different antipsychotics, of which at least 1 s generation antipsychotic, during at least 4 weeks in an adequate dosage. A list of adequate dosages of antipsychotic medication (see Appendix) was constructed using studies on comparable dosages of antipsychotics (21–23), information from the World Health Organization on Defined Daily Dosages (24) and Dutch guidelines (18). Three other indications for clozapine (markedly severe tardive dyskinesia, aggressive behavior or suicidality, all persisting during the use of two other antipsychotics) were also elaborated in the decision tree (5–11).

### Procedures

In June 2015, the psychiatrists and nurse practitioners of each team followed a training in the assessment of an indication for clozapine, during which the decision tree was introduced. After the training, the nurse practitioner identified all patients with NAPD by checking the DSM-IV codes, schizophrenia, schizophreniform disorder, schizoaffective disorder, or psychotic disorder not otherwise specified. The latter diagnosis was included because some Dutch psychiatrists are reluctant to use the word schizophrenia. In part of the teams, the controversy surrounding the concept of schizophrenia seems to have led to an increase of the diagnosis psychotic disorder not otherwise specified, and a lower use of schizophrenia as diagnostic label.

Patients with delusional disorder were not included, because clozapine has not shown to be effective for them. Those diagnosed with a brief psychotic disorder were also excluded because clozapine is not indicated for patients with this diagnosis.

The nurse practitioner of each team, assisted by the first author, reviewed the files of all the patients treated by that team. They identified the patients who were already on clozapine or had used this drug and had discontinued it. We assumed that all of these patients had an appropriate indication for this drug. Next, using the decision tree, the nurse practitioner, again assisted by the first author, assessed the remaining patients for clozapine indications, regardless of the feasibility of a trial with clozapine. These patients were divided into 3 groups: (a) no indication, (b) indication, and (c) questionable indication (for example a score of 4 “moderate” on the CGI-SCH or uncertainty about other indications or previous treatment with antipsychotics) Subsequently, they discussed the cases from the latter two categories with the responsible psychiatrist and tried to reach consensus about the indication for a trial with clozapine. In case of discordance, the opinion of the psychiatrist was decisive.

### Statistical analysis

Descriptive statistics were used to summarize demographic and clinical characteristics. χ^2^-tests were used to compare teams on all four types of patients.

After a Bonferroni correction for multiple testing, a two-tailed *p*-value of <0.008 was considered statistically significant for all tests.

## Results

### Demographic and clinical characteristics

In the FACT-teams, there were 2,286 patients with NAPD and in the early intervention teams 302 patients. The characteristics of these patients are presented in Table 1.

**Table 1:** Demographic and clinical features of 2,588 patients with Non-Affective Psychotic Disorder, treated by 20 Functional Assertive Community Treatment (FACT) teams and three Early Intervention teams in the Netherlands, 2016.

**Characteristic**	**Total**	**Clozapine, current users (Type 1)**	**Other patients**
**FACT teams**	***n* = 2,286**	***n* = 526**	***n* = 1,760**
	**Mean (SD)**	**Mean (SD)**	**Mean (SD)**
Age (years)	46.5 (11.6)	44.9 (10.7)	47.0 (11.9)
Sex, Male	65.9%	68.1%	64.4%
**DSM-IV DIAGNOSIS**	**(%)**	**(%)**	**(%)**
Schizophrenia (including schizophreniform disorder)	65.0	82.5	58.9
Schizoaffective disorder	15.2	12.9	15.8
Psychotic disorder not otherwise specified	19.8	4.6	25.3
**Early intervention teams**	***n* = 302**	***n* = 34**	***n* = 268**
	**Mean (SD)**	**Mean (SD)**	**Mean (SD)**
Age	33.8(10.1)	29.6(5.9)	34.2(10.4)
Sex, Male	68.9%	79.4%	67.7%
**DSM-IV DIAGNOSIS**	**(%)**	**(%)**	**(%)**
Schizophrenia (including schizophreniform disorder)	47.3	67.7	44.5
Schizoaffective disorder	6.0	8.8	5.9
Psychotic disorder not otherwise specified	46.7	23.5	49.6

### Use of clozapine

The overall actual clozapine prescription rate among FACT- and early intervention-teams (type 1 patients) was 21.6%. The overall proportion of patients with an indication for clozapine at any time type 1, type 2, and type 3) was 33.4% in FACT-teams and 18.2% in early intervention-teams (overall: 31.6%). Of these patients 68.3% was using clozapine (FACT-teams: 68.8% and early intervention-teams: 61.8%) also with a high variability between teams, see Table 2.

**Table 2:** Mean proportion and range of type 1, type 2, type 3, and type 4 patients and results of χ^2^-tests to compare teams, in 20 Flexible Assertive Community Treatment (FACT)-teams and 3 Early intervention teams in the Netherlands, 2016.

	**%**	**Range**	**χ^2^**	**df**	***p***
**FACT-teams *n* = 2,286**					
Type 1 (users)	23.0	8.8–34.7	53.6	19	<0.001^*^
Type 2 (former users)	3.5	0–8.2	26.75	19	0.110
Type 3 (unfulfilled indication)	6.9	1.7–15.6	55.97	19	<0.001^*^
Type 4 (as yet insufficiently treated)	8.6	1.8–16.3	52.29		<0.001^*^
Total of type 1, 2, and 3	33.4	17.6–47.6	66.97	19	<0.001^*^
Users among type 1, 2 and 3	68.8	49.0–90.9	46.64	19	<0.001^*^
**Early intervention teams *n* = 302**					
Type 1 (users)	11.3	8.5–14.1	1.35	2	0.509
Type 2 (former users)	2.3	1.1–3.6	1.88	2	0.391
Type 3 (unfulfilled indication)	4.6	0–9.4	13.14	2	0.001^*^
Type 4 (as yet insufficiently treated)	11.6	7.0–16.3	3.51	2	0.171
Total of type 1, 2, and 3	18.2	11.3–23.7	5.70	2	0.058
Users among type 1, 2, and 3	61.8	45.5–92.9	10.04	2	0.007

* *A p-value of < 0.008 was considered statistically significant*.

**Figure 1:**
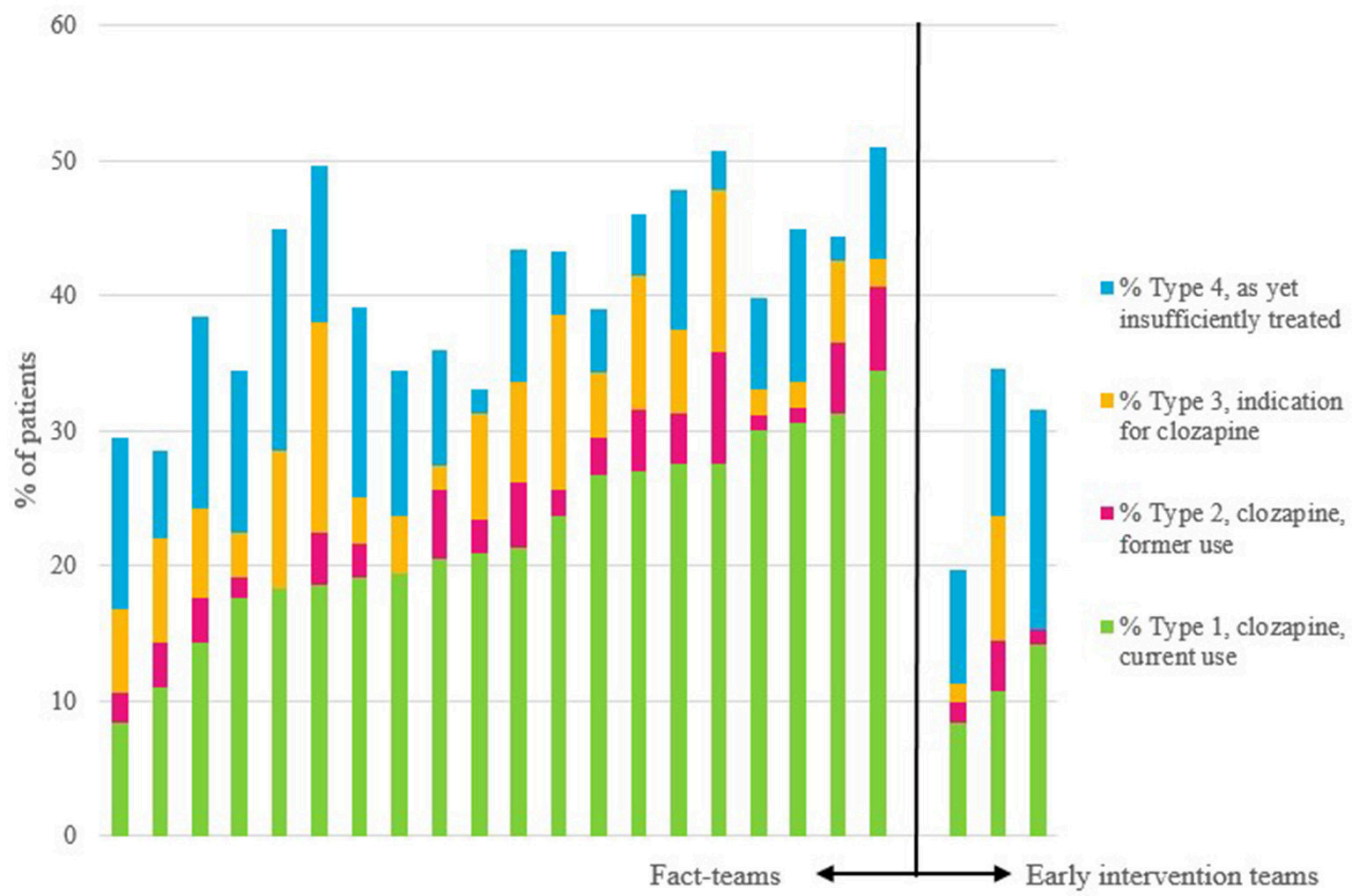
Per team: proportions of current users of clozapine, of previous users, of those with an indication for this drug, and of those as yet insufficiently treated to have a diagnosis of treatment-resistance. The first 20 bars are the FACT-teams, and the last three bars are the early intervention teams.

The proportions of type 1, type 2, type 3, and type 4 patients and the ranges between teams are shown in Table 2, by type of team. We found a significant variability between teams with regard to the proportions of all 4 types of patients (see Figure 1). The overall rate of underprescription (type 3 patients) was 6.6%. In 94.8% of these patients treatment-resistant positive symptoms were the reason for the clozapine indication (see for indications and combinations of indications Table 3). Only 5.2% of patients had other indications without treatment-resistant positive symptoms.

**Table 3:** Type of indication for clozapine among 172 patients with Non-Affective Psychotic Disorder, with an unfulfilled indication, from 20 Flexible Assertive Community Treatment (FACT)-teams and 3 early intervention teams in the Netherlands, 2016.

**Single indication *N* = 134**	***N***	**%**
Treatment-resistant positive symptoms	125	72.7
Aggression	1	0.6
Suicidality	3	1.7
Acute extrapyramidal symptoms	3	1.7
Tardive dyskinesia	2	1.2
**Multiple indications *N* = 38**		
**TREATMENT-RESISTANT POSITIVE SYMPTOMS**		
And aggression	24	14.0
And suicidality	8	4.7
And suicidality + aggression	1	0.6
And extrapyramidal symptoms	2	1.2
And tardive dyskinesia	1	0.6
And extrapyramidal symptoms + tardive dyskinesia	2	1.2

## Discussion

### Main findings

The prescription rate of clozapine in FACT- and early intervention-teams was 21.6% (type 1 patients), and the rate of underprescription was 6.6% (type 3). However, the latter proportion is probably higher, because a part of the as yet insufficiently treated patients (type 4) may turn out to have an indication for this drug too. The differences between teams in prescribing and underprescribing clozapine, were very large and statistically significant.

### Interpretation

The proportion of outpatients with NAPD on clozapine in this study is higher than those reported by other European studies in outpatients. A national database study in Denmark found that 10.1% of the patients with a diagnosis of schizophrenia was using clozapine (15). In a study in France, only 1.2% of the users of antipsychotics was using clozapine. However, 6.6% of the total population was using antipsychotics, so the clozapine prescription rate among patients with NAPD may be much higher (13). Although different study designs may contribute to these differences, a large international database study (25) also showed that prescription rates in the Netherlands are higher than in most other countries. A national clozapine expertise center, founded in 2004, may have contributed to this Bogers et al. (26). An audit in the UK, with a comparable real-world design found similar rates of 23.7% (27). However, Patel et al. only included patients that were under care for at least 12 months, which may have led to somewhat higher clozapine prescription rates. They found a proportion of 21.2% of patients who were not or partially in remission (no definition given) and were not prescribed clozapine. Sixty-one percent of these patients had already received two adequate trials of antipsychotics, suggesting that 12.9% was having an unfulfilled indication for clozapine. However, drug adherence had been investigated for only 85% of them.

We found considerable difference in clozapine prescription rates between teams which may partly be caused by differences in the severity of psychopathology. However, we also found significant differences in prescription rates after restricting the analysis to patients with an indication for this drug. It is unlikely therefore, that the variability between teams is entirely explained by differences in severity of psychopathology. Other explanatory variables are local norms and traditions (13, 28, 29) or specific clinician-related factors, such as their workload, knowledge and preferences (30). The more the time of the psychiatrist is restricted, the more difficult it may become to supervise the weekly blood drawings and monitor adequately for potentially lethal side-effects. Consequently, extra staff, such as the deployment of a nurse practitioner, may help in preventing needless delay in clozapine initiation (31).

### Strengths and limitations

A strength of this study is the large number of patients from both FACT- and early intervention-teams from four different institutes. Additionally, we were able to determine the exact proportion on clozapine and the decision tree allowed for a standardized method to assess indications for clozapine. However, several limitations require comment. First, the teams of this study belonged to institutes that had agreed to participate in a trial on the safety of the deployment of nurse practitioners to start patients on clozapine. Consequently, the non-random selection of teams diminishes the generalizability of the results. Second, the quality of the information obtained from electronic files was not optimal in all cases. Some diagnoses may have been inaccurate and some information on antipsychotics was lost during the transition from paper file to electronic file, about 10 years ago. Information on the presence of tardive dyskinesia was often lacking and was almost solely obtained verbally from the responsible nurse practitioner or psychiatrist. Third, the rating of the severity of the positive symptoms may not always have been perfectly valid. However, since there were only 2 cases of discordance on the indication for clozapine, the decisive role of the psychiatrist barely influenced the results. Finally, our definition of treatment-resistance differed somewhat from that in recently published guidelines (2), in that it was based on a higher score for the severity of positive symptoms (marked instead of moderate), on a adherence of 90% of prescribed antipsychotics taken (instead of 80%), but on a shorter duration of adequate treatment (4 instead of 6 weeks) and on a slightly lower minimum dosage of antipsychotic drug, see Appendix. Consequently, a substantial over- or underestimation of the number of indications for clozapine is highly unlikely.

## Conclusion

In conclusion, about a third of the Dutch outpatients with NAPD is indicated for the use of clozapine and more than two-thirds of them are using it. By international standards, the clozapine prescription rates in Dutch ambulatory care are high, but the differences between teams are considerable. Research into reasons for this variability is urgently needed to develop targeted interventions.

## Ethics statment

This study complies with the ethical rules for human experimentation as stated in the Declaration of Helsinki and participation was voluntary. The study was approved by the Medical Ethics Committee of the RTPO (Regionale Toetsingscommissie Patiëntgebonden Onderzoek), Leeuwarden, The Netherlands.

## Author contributions

JS, PS, and YvdZ designed the study and wrote the protocol. FT and YvdZ undertook the statistical analysis, and author YvdZ wrote the first draft of the manuscript. All authors contributed to and have approved the final manuscript.

### Conflict of interest statement

The authors declare that the research was conducted in the absence of any commercial or financial relationships that could be construed as a potential conflict of interest.
